# Ultrasound-Guided Cannulation: Time to Bring Subclavian Central Lines Back

**DOI:** 10.5811/westjem.2016.1.29462

**Published:** 2016-03-02

**Authors:** Talayeh Rezayat, Jeffrey R. Stowell, John L. Kendall, Elizabeth Turner, J. Christian Fox, Igor Barjaktarevic

**Affiliations:** * David Geffen School of Medicine, UCLA, Division of Pulmonary and Critical Care Medicine, Los Angeles, California; † Maricopa Medical Center, Department of Emergency Medicine, Phoenix, Arizona; ‡ Denver Health Medical Center, Department of Emergency Medicine, Denver, Colorado; § University of California, Irvine, Department of Emergency Medicine, Irvine, California

## Abstract

Despite multiple advantages, subclavian vein (SCV) cannulation via the traditional landmark approach has become less used in comparison to ultrasound (US) guided internal jugular catheterization due to a higher rate of mechanical complications. A growing body of evidence indicates that SCV catheterization with real-time US guidance can be accomplished safely and efficiently. While several cannulation approaches with real-time US guidance have been described, available literature suggests that the infraclavicular, longitudinal “in-plane” technique may be preferred. This approach allows for direct visualization of needle advancement, which reduces risk of complications and improves successful placement. Infraclavicular SCV cannulation requires simultaneous use of US during needle advancement, but for an inexperienced operator, it is more easily learned compared to the traditional landmark approach. In this article, we review the evidence supporting the use of US guidance for SCV catheterization and discuss technical aspects of the procedure itself.

## INTRODUCTION

Since its original description over 60 years ago by Aubaniac, the subclavian vein (SCV) has been an important vessel for central venous cannulation.^1^ The SCV cannulation offers several advantages when compared to the common alternative sites for central venous access. These advantages may include fewer cases of thrombosis, infectious complications, better patient comfort, and increased ability to remain patent in hypovolemic states.^2^–^8^ Unfortunately, cannulation of the SCV is not without complications such as catheter malposition, arterial puncture, hematoma, pneumothorax, hemothorax, and nerve injury. The rate of clinically relevant mechanical complications has been shown to be as high as 18.8%, likely due to the traditional landmark (LM)-guided or “blind” approach, and also dependent on user experience.^4^,^6^ As a result, alternative approaches to SCV cannulation, including ultrasound (US)-guided techniques, have been explored and determined to have improved safety and reduced complications particularly when using real-time US in the longitudinal or “in-plane” method.^9^,^10^ In this article, we review the evidence supporting the use of US guidance for SCV catheterization, and discuss technical aspects of several approaches.

## ULTRASOUND GUIDANCE

Wide availability and improved technology have made bedside US a valuable tool for establishing vascular access. It allows for direct visualization of and evaluation for the vessel of choice in addition to precise needle positioning during cannulation.^11^,^12^ Multiple studies have compared US-guided central vein catheterization to LM techniques and found US superior with a 12% reduction of unsuccessful line placement, 1.19 fewer attempts, and a 71% reduction in overall catheter-related complications for internal jugular vein (IJV) placement.^9^,^13^ As a result, multiple national and international organizations, including The American College of Emergency Physicians (ACEP), National Institute for Clinical Excellence (NICE), and the Agency for Healthcare Research Quality (AHRQ), recommend the use of US in central vein cannulation.^14^,^15^

At first glance, the SCV seems difficult to visualize on US because it travels beneath the highly reflective clavicle bone. This, along with the higher complication rate of the LM-guided approach, has resulted in the SCV falling out of favor for elective central vein cannulation in many modern clinical settings. To explore this notion, recent studies have compared the use of US to the LM approach in SCV cannulation (Table) and results suggest a significant impact of US on the safety and feasibility of SCV cannulation. In a prospective randomized control trial by Fragou et al comparing real-time US guidance with the LM technique, US guidance was found to improve success rates, 100% vs. 87.5% and reduce the rate of mechanical complications, including arterial puncture and hematoma formation.^10^ Additionally, there was a reduction in the rate of pneumothorax (4.9%) using US, likely secondary to the ability to visualize the needle and prevent posterior vessel wall penetration.^10^,^16^ Two recent meta-analyses also showed a significant reduction in arterial puncture and hematoma formation, as well as improved rate of successful cannulation when using real-time US with a longitudinal “in-plane” infraclavicular approach.^9^,^17^ Similarly, Randolph et al demonstrated the use of US was associated with a reduced risk of catheter placement failure (relative risk 0.32; 95% confidence interval 0.18 to 0.55), lower overall complication rates (relative risk 0.22; 95% confidence interval 0.10 to 0.45), and a reduced number of needle sticks before successful placement (relative risk 0.60; 95% confidence interval 0.45 to 0.79), for both SCV and IJV cannulation.^11^ Gualtieri et al demonstrated that the use of US improved the SVC cannulation success rate in less-experienced operators (92% vs 44%).^16^ The benefits of US guidance make the SCV an excellent option for central venous cannulation.

## ULTRASOUND TECHNIQUES: LONGITUDINAL VS*.* SHORT AXIS VIEW

**Figure 1:**
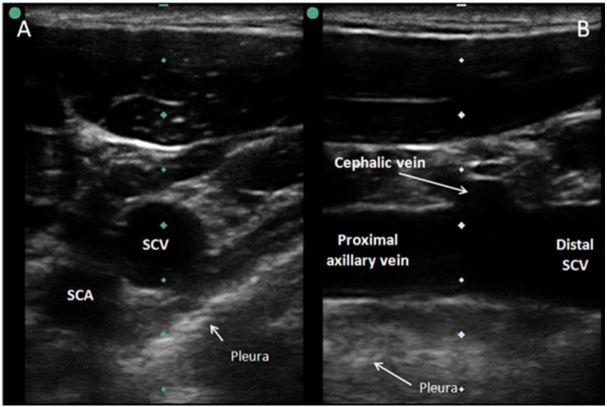
A) Short axis view of subclavian vein using ultrasound vascular probe. B) Long axis view of subclavian vein using ultrasound vascular probe. *SCV*, subclavian vein; *SCA*, subclavian artery

Positioning the long footprint of the US probe perpendicular to the course of the vessel gives rise to a short axis view (Figure 1a). This view allows for visualization of the target vessel and surrounding structures, and offers the operator a good midline orientation. This view allows for an “out-of-plane” needle-guided approach, which does not offer the optimal ability to visually control the needle tip during the cannulation process. This is because the needle artifact on the screen only shows a cross section of the needle. This may be the needle tip, but it could also be any part of the needle shaft – they look identical on US. Alternatively, the longitudinal, or long axis view, is obtained with the transducer and vessel axes in parallel (Figure 1b). This view identifies the target vessel along its length. Using this view for obtaining vascular access allows one to insert the needle using an “in-plane” needle tip approach which allows for direct and full visualization of both the needle tip and needle shaft during catheterization. The needle is easily witnessed entering the target vessel and, importantly, the guidewire’s direction of travel can be verified. The challenge with the “in-plane” technique requires the operator to have the dexterity needed to line up the one millimeter thickness of sound beam with the one millimeter thickness of needle, all within the midline axis of the vessel’s longitudinal plane. Another potential limitation of the long axis approach is not being able to simultaneously see both artery and vein on the screen as in the short axis approach. After identification of the vein in the long axis, it is possible that due to necessary coupling gel, that the operator’s hand could slide a few millimeters and be visualizing the artery. In the long axis veins and arteries can appear similar, particularly when they are in an area that is not conducive to compression.

A single-center randomized crossover control trial including 57 emergency medicine residents and attending physicians of varying US experience compared the short axis versus long axis approach for axillary vein cannulation using a torso phantom model.^18^ The long axis approach was superior for successful placement on initial attempt with fewer needle redirections and reduced complications. When surveyed, the long axis approach was also the preferred approach of the examined operators. In another prospective study comparing emergency medicine trainees’ skills in obtaining an adequate view for catheterization using a human torso model, the long axis SCV view led to quicker access time, reduced redirections, and significantly fewer posterior wall penetrations compared to the short axis probe orientation.^16^

## SUPRACLAVICULAR SUBCLAVIAN VEIN CANNULATION UNDER ULTRASOUND GUIDANCE

Multiple studies have demonstrated the advantages of a supraclavicular approach to SCV cannulation, but results have been dependent on operator experience.^19^,^21^ The approach has a well-defined insertion LM - the clavisternomastoid angle, with insertion from above the clavicle.^21^ This approach offers a shorter, more direct course to the SCV, traversing only fascial planes, whereas with an infraclavicular approach, it must traverse the pectoralis major muscle, which may lead to increased catheter malpositioning.^22^,^23^ In a randomized prospective comparative study of infraclavicular vs. supraclavicular approaches using a LM technique, there was a 9% incidence of catheter malpositioning in the infraclavicular group compared to 0.5% in the supraclavicular group.^22^ In another prospective comparative study evaluating 144 patients requiring central venous catheterization, a supraclavicular approach had a statistically significant higher success rate in comparison to an infraclavicular approach.^20^

There are limited published data comparing supraclaviclar to infraclavicular approaches with real-time US guidance. In one prospective anatomical study of normovolemic patients, Stachura et al demonstrated that identifying the SCV in the supraclavicular region using US is technically easier compared to the infraclavicular region.^8^ The use of real-time US for supraclavicular SCV cannulation is limited by a lack of space in the supraclavicular area for both the US probe and the needle used for cannulation.^22^ Understanding this limitation, Mallin et al described a supraclavicular approach using an endocavitary probe with a smaller footprint, creating adequate space for real-time US-guided cannulation.^24^ As most US systems are not routinely equipped with endocavitary probes, it is not surprising that currently available literature favors the infraclavicular approach as the preferred approach for SCV cannulation. Future studies focused on smaller US vascular probes may lead to better understanding of the value of the supraclavicular approach.

## INFRACLAVICULAR SUBCLAVIAN CANNULATION UNDER ULTRASOUND GUIDANCE

The axillary vein courses medially and becomes the SCV at the lateral border of the first rib. It continues its path under the clavicle, arching upward across the superior surface of the first rib and then inclines medially, downwards and across the insertion of the anterior scalene muscle. At this point, it enters the thorax as it unites with the IJV behind the sterno-clavicular joint.^10^,^21^,^25^ SCV visualization via US is possible in the clavipectoral triangle, 2–3cm distal to the point where the SCV crosses below the clavicle. As a result, US-guided SCV cannulation using an infraclavicular approach is positioned near the border of the axillary vein, which is noticeably lateral to the LM approach.^26^,^27^

**Figure 2:**
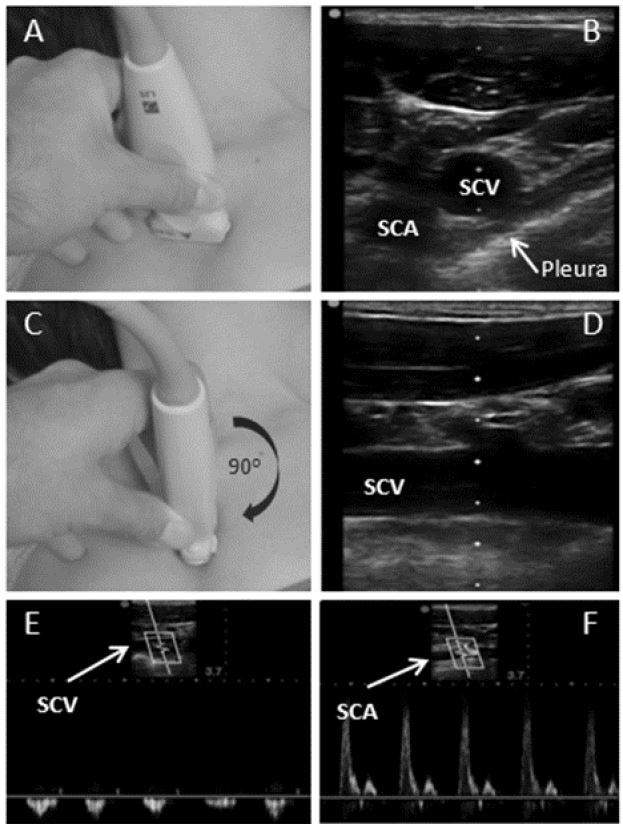
A) Linear transducer is placed perpendicularly and inferior to clavicle. B) Identified anatomical structures include the transverse (short axis) view of subclavian vein (SCV), subclavian artery (SCA) and pleura. C) With SCV centrally positioned, the transducer is rotated 90° clockwise until D) longitudinal view of subclavian vein is obtained. E) Pulse-wave Doppler view of the SCV confirms non-pulsatile flow and identifies the vessel. F) Tilting the transducer cephalad enables the visualization and identification of SCA with pulse-wave Doppler ultrasound for better anatomic orientation.

**Figure 3:**
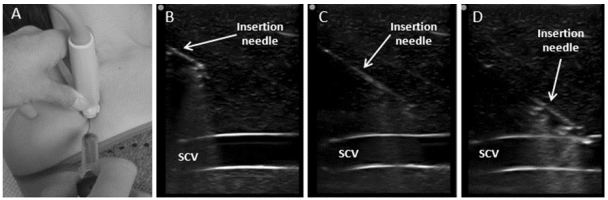
A) After identification and in-plane alignment of subclavian vein (SCV) on ultrasound, the insertion needle enters the skin at midpoint of the transducer’s small footprint and is advanced within the plane of ultrasound penetration. B), C) and D) The transducer remains in steady position enabling continuous longitudinal view of SCV, and the needle is carefully and slowly introduced with maintenance of needle visualization until the anterior wall of SCV is punctured.

The procedure begins with the patient placed in a supine position, prepared and draped in a sterile fashion. The subclavian and axillary veins are visualized by placing a high frequency linear transducer in the infraclavicular fossa (Figure 2a), in order to obtain a short axis view of the vein and artery (Figure 2b). After identification of the target vessel, the vein is positioned centrally on the screen and the transducer is rotated (Figure 2c), maintaining visualization of the vein, until a longitudinal view is obtained. This view enables visualization of axillary vein and distal SCV, as well as the pleural lining below the vessel (Figure 2d). Tilting the transducer cephalad enables visualization of the subclavian artery, and is used to identify and differentiate the vein from the artery. Vessel compressibility and venous pattern on pulse-wave Doppler are generally recommended for confirmation of the appropriate vessel (Figure 2e and 2f). In the longitudinal orientation, the needle is inserted in the midpoint of the small footprint of the transducer (Figure 3a), enabling an in-plane view. The inserting needle should be advanced slowly and visualized throughout the entire procedure while maintaining a view of the vessel and adjacent anatomical structures (Figure 3b–d). If needle visualization is lost, it is essential to avoid complications by ceasing to advance the needle, withdrawing slightly and then relocating the needle tip and shaft before proceeding. Once within the lumen of the vessel the guidewire is inserted with the J-tip pointing caudad and the direction of travel visualized in real time. The anticipated length of line insertion is, in general, 1–2cm longer in comparison to the length anticipated with subclavicular LM approach due to the more lateral approach described above.

This longitudinal, real-time, US-guided infraclavicular SCV cannulation approach offers several advantages to the LM technique. Using this approach, the operator can control the advancement of the needle, identify adjacent anatomical structures, including the pleura and posterior wall of the vessel. This in turn allows for a decreased risk of posterior vessel wall puncture, lowering the subsequent risk of pneumothorax.^16^ Additionally, the approach has been demonstrated to decrease the rate of arterial puncture and hematoma formation.^9^,^17^ Additionally, real-time longitudinal views lead to a significantly increased overall success rate, with fewer attempts, redirections, or malpositioned catheters.^10^,^16^,^25^ In a prospective study by Fragou et al., 401 sedated and mechanically ventilated patients were randomized to either real-time US guidance (n=201) or LM technique (n=200) for placement of subclavian catheters by experienced operators.^10^ This study found the time to obtain vascular access and number of attempts were significantly lower using real-time US guidance (p<0.05). It is, however, possible that with an inexperienced operator or due to US preparation time, US-guided line placement may be slightly longer in duration in comparison to LM approach.^25^ Lastly, SCV cannulation can be learned on simulation models more rapidly with US guidance compared to the LM technique. In a study by Tokumine et al., 20 medical trainees received instruction on both LM and US-guided SCV cannulation using the longitudinal axis.^23^ Sufficient skill to place an US-guided SCV catheter was achieved with three attempts compared to nine for the LM technique.

## CONCLUSION

The SCV offers multiple advantages as a target for central venous access in the appropriately selected patient. The use of real-time US guidance for infraclavicular placement of SCV catheters allows for direct visualization of needle insertion and adjacent anatomical structures, as well as guidewire location and directionality, all of which can lead to decreased mechanical complications and improved cannulation success, compared to a LM technique. Although more research is needed, in our opinion the current literature supports the use of the infraclavicular longitudinal US-guided SCV catheterization as the preferred technique for cannulation of SCV when compared to LM approach and a solid alternative to cannulation of IJVs.

## Figures and Tables

**Table:** Studies evaluating direct ultrasound-guided subclavian vein cannulation in comparison to landmark approach.

Authors/publication	Type of study	Participants	Enrollment	Operators	Outcomes
Fragou et al. 10	Prospective randomized single center	Mechanically ventilated and sedated patients in the medical ICU	LM group: N=201, US group: N=200	Multiple, with more than 6 years of experience in placement of central venous catheters	Increased success rate for experienced operators (100% vs 87.5%) Significantly decreased mechanical complication rate
Alic, Y et al. 28	Prospective randomized single center	ICU patients (type of ICU not specified)	LM group: N=35, US group: N=35	One physician experienced in both techniques	No significant difference between success at 1^st^ attempt, overall success, or complication rate between LM and US group.
Palepu et al. 29	Prospective randomized single center	Combined medical and surgical ICU Patients	LM group: N=28, US group: N=17	Multiple operators with varying levels of experience	No significant difference between overall success (p=0.52), number of attempts (p=0.23) or complication rate (p>0.99)
Gualtieri et al. 30	Prospective randomized single center	Combined trauma, surgical and medical ICU Patients	LM group: N=27, US group: N=25	More than one operator with varying levels of experience	Increased success rate for inexperienced operators (92% vs 44%) using direct US guidance Reduced minor complications (4% vs 41%)

ICU, intensive care unit; LM, landmark; US, ultrasound

## References

[1] Aubaniac R (1952). Subclavian intravenous injection; advantages and technic. La Presse medicale.

[2] Marik PE, Flemmer M, Harrison W (2012). The risk of catheter-related bloodstream infection with femoral venous catheters as compared to subclavian and internal jugular venous catheters: a systematic review of the literature and meta-analysis. Crit Care Med.

[3] McGee DC, Gould MK (2003). Preventing complications of central venous catheterization. New Engl J Med.

[4] Merrer J, De Jonghe B, Golliot F (2001). Complications of femoral and subclavian venous catheterization in critically ill patients: a randomized controlled trial. JAMA.

[5] O’Grady NP (2012). Zero risk for central line-associated bloodstream infections ... Is this realistic?. Crit Care Med.

[6] Ruesch S, Walder B, Tramer MR (2002). Complications of central venous catheters: internal jugular versus subclavian access--a systematic review. Crit Care Med.

[7] Shah ASA, Panchatsharam S (2013). Ultrasound-guided subclavian venous catheterisation - is this the way forward? A narrative review. Int J Clin Pract.

[8] Stachura MR, Socransky SJ, Wiss R (2014). A comparison of the supraclavicular and infraclavicular views for imaging the subclavian vein with ultrasound. Am J Emerg Med.

[9] Brass P, Hellmich M, Kolodziej L (2015). Ultrasound guidance versus anatomical landmarks for subclavian or femoral vein catheterization. Cochrane Database Syst Rev.

[10] Fragou M, Gravvanis A, Dimitriou V (2011). Real-time ultrasound-guided subclavian vein cannulation versus the landmark method in critical care patients: a prospective randomized study. Crit Care Med.

[11] Randolph AG, Cook DJ, Gonzales CA (1996). Ultrasound guidance for placement of central venous catheters: a meta-analysis of the literature. Crit Care Med.

[12] Timsit JF (2003). What is the best site for central venous catheter insertion in critically ill patients?. Crit Care.

[13] Miller AH, Roth BA, Mills TJ (2002). Ultrasound guidance versus the landmark technique for the placement of central venous catheters in the emergency department. Acad Emerg Med.

[14] National Institute for Clinical Excellence (2002). NICE technology appraisal guidance No 49: guidance on the use of ultrasound locating devices for placing central venous catheters.

[15] American College of Emergency P (2006). Emergency ultrasound imaging criteria compendium. American College of Emergency Physicians. Ann Emerg Med.

[16] Vogel JA, Haukoos JS, Erickson CL (2014). Is Long-Axis View Superior to Short-Axis View in Ultrasound-Guided Central Venous Catheterization?. Crit Care Med.

[17] Lalu MM, Fayad A, Ahmed O (2015). Ultrasound-Guided Subclavian Vein Catheterization: A Systematic Review and Meta-Analysis. Crit Care Med.

[18] Sommerkamp SK, Romaniuk VM, Witting MD (2013). A comparison of longitudinal and transverse approaches to ultrasound-guided axillary vein cannulation. Am J Emerg Med.

[19] Byon HJ, Lee GW, Lee JH (2013). Comparison between ultrasound-guided supraclavicular and infraclavicular approaches for subclavian venous catheterization in children--a randomized trial. Brit J Anaesthes.

[20] Hussain S, Ahmad Khan R, Iqbal M (2011). A comparative study of supraclavicular versus infraclavicular approach for central venous catheterization. Anaesth, Pain & Intens Care.

[21] Yoffa D (1965). Supraclavicular subclavian venepuncture and catheterisation. Lancet.

[22] Sterner S, Plummer DW, Clinton J (1986). A comparison of the supraclavicular approach and the infraclavicular approach for subclavian vein catheterization. Ann Emerg Med.

[23] Tokumine J, Matsushima H, Lefor AK (2014). Ultrasound-guided subclavian venipuncture is more rapidly learned than the anatomic landmark technique in simulation training. J Vasc Access.

[24] Mallin M, Louis H, Madsen T (2010). A novel technique for ultrasound-guided supraclavicular subclavian cannulation. Am J Emerg Med.

[25] Oh AY, Jeon YT, Choi EJ (2014). The influence of the direction of J-tip on the placement of a subclavian catheter: real time ultrasound-guided cannulation versus landmark method, a randomized controlled trial. BMC Anesthesiology.

[26] Sandhu NS (2004). Transpectoral ultrasound-guided catheterization of the axillary vein: an alternative to standard catheterization of the subclavian vein. Anesth Analg.

[27] Stefanidis K, Fragou M, Pentilas N (2012). Optimization of Cannula Visibility during Ultrasound-Guided Subclavian Vein Catheterization, via a Longitudinal Approach, by Implementing Echogenic Technology. Crit Care Res Pract.

[28] Alic YTAaPA (2009). Ultrasound-guided catheterization of the subclavian vein: a prospective comparison with the landmark technique in ICU patients. Crit Care.

[29] Palepu GB, Deven J, Subrahmanyam M (2009). Impact of ultrasonography on central venous catheter insertion in intensive care. Indian J Radiol Imaging.

[30] Gualtieri E, Deppe SA, Sipperly ME (1995). Subclavian venous catheterization: greater success rate for less experienced operators using ultrasound guidance. Crit Care Med.

